# ﻿Typification of five plant names described based on specimens collected by Józef Warszewicz in Central and South America

**DOI:** 10.3897/phytokeys.192.78409

**Published:** 2022-03-10

**Authors:** Marcin Nobis, Ewelina Klichowska, Mateusz Wolanin, Agnieszka Nobis, Arkadiusz Nowak

**Affiliations:** 1 Institute of Botany, Jagiellonian University, Gronostajowa 3, 30–387 Kraków, Poland Jagiellonian University Kraków Poland; 2 Institute of Biology and Biotechnology, Rzeszów University, Zelwerowicza 4, 35–601 Rzeszów, Poland Rzeszów University Rzeszów Poland; 3 Institute of Biology, University of Opole, 45–052 Opole, Poland University of Opole Opole Poland; 4 Botanical Garden, Center for Biological Diversity Conservation, Polish Academy of Sciences, 02–976 Warszawa, Poland Center for Biological Diversity Conservation, Polish Academy of Sciences Warszawa Poland

**Keywords:** *Berberis*, *Esenbeckia*, KRA herbarium, lectotypification, neotropics, plant hunter, *Proclesia*, *Psammisia*, *Remijia*, *Rondeletia*, Warscewicz

## Abstract

Józef Warszewicz (1812–1864) was one of the first Polish naturalists to explore the flora of the tropical New World. During two expeditions to Central and South America (1844–1850 and 1850–1853) he collected and delivered to Europe up to twenty thousand plant specimens. To honour his service and his achievements in plant collections, different taxonomists described more than 100 taxa using the surname Warszewicz, for example in the genus name (*Warszewiczia*) and the species epithets (*warszewiczii*, *warscewiczii*, *warszewicziana*). Unfortunately, a large part of Warszewicz’s collection of plant species deposited in the Berlin Herbarium (B), including many type specimens was destroyed during World War II. During digitisation of herbarium collections preserved in the Herbarium of the Jagiellonian University (KRA), we reviewed more than 650 herbarium sheets with plant specimens collected by Warszewicz and originating from his trips to Central and South America. In this paper, we present the typification of five names of species, described base on Warszewicz’s plant material. We select lectotypes for *Berberiswarszewiczii*, *Esenbeckiawarscewiczii*, *Psammisiaramiflora*, *Remijiainvolucrata* and *Rondeletiaorthoneura*, and provide data on the presence of 17 specimens (isotypes) representing *Esenbeckiacornuta*, an extremely rare species, that to date is known only from the type locality in Peru. A list of all Warszewicz’s specimens preserved at KRA herbarium is also presented. Additionally, in the result of the revision of syntypes of *Berberismultiflora* and *Rondeletiareflexa* we designated here the lectotypes for these taxa.

## ﻿Introduction

Józef Warszewicz (1812–1864) known also as ‘Josef Ritter von Rawicz Warszewicz or Josef Ritter von Warszewicz’ (sometimes misspelled e.g. as Warscewicz or Warczewicz), was one of the first Polish naturalists who had the opportunity to explore the flora of Central America. Having Aleksander von Humboldt’s recommendation letter, Warszewicz joined an expedition of the Belgian Horticultural Society to Guatemala in 1844. The expedition was organised to collect plant seeds, learn about natural resources of the area, and obtain knowledge on the possibilities of its colonisation. The expedition started tragically since all its participants got sick and except for Warszewicz and his colleague doctor Flemish, died of yellow fever. Nevertheless, after recovering, Warszewicz set off alone to explore the area (Zemanek 2013; Köhler 2014; Jungfer 2017; Zemanek et al. 2021). His first stay in Central America lasted from 1844 to 1850, but the exact route of these peregrinations is difficult to determine. He probably travelled from Guatemala, through Honduras and Nicaragua to Costa Rica and Panama (Savage 1970; Sauter 2010; Zemanek 2013; Jungfer 2017; Nobis et al. 2020a). Warszewicz collected mostly plant seeds, seedlings as well as living and dried plant specimens. Initially, his main recipient and sponsor was Luis van Houtte (1810–1876) associated with the Botanic Garden in Brussels, however, Warszewicz also sent plants to many public and private botanical gardens (mainly in Berlin, Hamburg, London and Zurich) which provided him the funds to continue the expedition. He collected also some animal specimens such as snails, amphibians, reptiles and birds for natural history museums. Warszewicz returned to Europe at the beginning of 1850 and stayed in Berlin for several months to work as a private assistant to Heinrich Gustav Reichenbach (1823–1889). Although Warszewicz was not a professional taxonomist, he described, in cooperation with Reichenbach, many new orchid species. Afterward, at the end of 1850, he set off on a second expedition to Central and South America (1851–1853). This time he explored vast areas of Panama, Ecuador, Peru, Bolivia and Brazil. The botanical collection made by Warszewicz was impressive. He delivered to Europe up to twenty thousand specimens of plants. The collection included several hundred new taxa, especially orchids (Orchidaceae, about 300 taxa), but also cycads (Cycadopsida), cannas (Cannaceae) and other groups of tropical plants (Jungfer 2017; Zemanek et al. 2021). Based on his collections, many new species were described by German taxonomists, such as Johann Friedrich Klotzsch (1805–1860), Heinrich Gustav Reichenbach and Eduard August von Regel (1815–1892). To honour his service and his achievements in plant collections, over 100 taxa were described by different researchers using Warszewicz’s surname (*Warszewiczia*, *warszewiczii*, *warscewiczii*, *warszewicziana*; IPNI 2021). Unfortunately, a large part of Warszewicz’s collection deposited at Berlin herbarium (B) including original specimens (holotypes and syntypes) was destroyed during World War II on the night of 1–2 March 1943. Fortunately, some of the types, or their duplicates, have survived and today are preserved in the herbaria of B, G, BR, K, BM, P, LD, F, NY and KRA (acronyms follow Thiers 2021).

During the digitisation process of the collections preserved in the Herbarium of the Jagiellonian University (KRA), we reviewed Warszewicz’s plant collections, originating from his trips to Central and South America. More than 650 herbarium sheets with specimens collected by him are preserved as a separate collection (all the sheets have been scanned and can be shared by request to the first author). In KRA, all the herbarium specimens collected by Warszewicz have the same label “*Reliquiae Warszewiczianae*, *America merid: Columbia. lg. J. Warszewicz*” (printed and attached to this collection at the beginning of 20^th^ century by Antoni Żmuda). However, to selected specimens/gatherings, the labels with original Warszewicz’s collection number and place of the collection are attached as well. It is worth noting that a number of Warszewicz’s specimens preserved in B, also did not have any information about the date and place of collection (see Berlin negatives at the F herbarium online: https://collections-botany.fieldmuseum.org/list). Specimens from Warszewicz’s collection deposited in KRA were studied and determined by Stanisław Kulczyński and Antoni Żmuda from the Institute of Botany, Jagiellonian University in Kraków in 1915. However, they were able to identify only a part of this collection, and numerous specimens still remained undetermined. Most of Warszewicz’s collection is represented by specimens of trees and shrubs (e.g.: *Alnus* Mill., *Bejaria* Mutis, *Berberis* L., *Cinchona* L., *Esenbeckia* Knuth, *Lonicera* L., *Morus* L., *Nectandra* Rolander ex Rottbøll, *Petrea* L., *Pernettya* Gaudichaud-Beaupré, *Salix* L., *Warszewiczia* Klotzsch, *Weigeltia* A. DC.). Although Warszewicz was focused mainly on ornamental plants that could be adopted in European botanical gardens, in his collection there are also several herbaceous plants without much ornamental value (e.g., species of *Gentiana* L., *Lobelia* L., *Polygala* L., *Utricularia* L., *Vernonia* Schreb.). Warszewicz’s collection preserved in KRA includes original specimens (duplicates) of the type material that was preserved at B herbarium but destroyed during the WWII. Fortunately, J. Francis Macbride documented on photographs most of the types preserved in B before WWII (Hiepko 1987; Nascimento et al. 2019; BGBM 2020), which nowadays can be used during comparative taxonomical studies. However, because we cannot be sure that Macbride photographed all duplicates of the type materials (Nascimento et al. 2019; BGBM 2020), the photographs cannot be considered as evidence that only one specimen of a particular gathering was originally present in B. Following the ICN (Turland et al. 2018) the photographs of original material have no nomenclatural standing, since they were not available to the author prior to being cited or published in the protologue, and therefore, cannot be designated as lectotypes. However, they can be designated as neotypes, standing in place of the destroyed Berlin specimens, if no other original material survives (Art. 9.8 in Turland et al. 2018). A detailed discussion on the nomenclatural value of photographs of type specimens, with a number of examples of how photographs were treated by different taxonomists has been presented by Staples and Prado (2018) and Nascimento et al. (2019), with some suggestions on future ICN improvements.

Thanks to the digitisation of herbarium collections, images of sheets with plant specimens are available on websites of many herbaria, making them available to a wide audience. Herbaria provide material samples for biological research in diverse fields (Funk 2003), including global change biology (Meineke et al. 2018; Lang et al. 2019), and are an important source of species discovery (Bebber et al. 2010). They play an integral role in a modern additive research process (Henning et al. 2018) that aims to describe and understand the evolution and diversity of organisms worldwide (Harris and Marsico 2017; Borsch et al. 2020). Easily accessible and searchable online data on herbarium specimens definitely increase research efficiency. Time previously spent travelling to collections or waiting for specimen loans could instead be spent on gathering data. Taxonomists are often able to use digitised collections to identify and annotate specimens (Harris and Marsico 2017), to indicate typical specimens and to typify names of species for which the type has not been designated or are regarded as lost or destroyed. In recent years, publications including typification made on the basis of available online herbarium databases are becoming more and more common (e.g. Sukhorukov et al. 2019; Alves-Araújo et al. 2020; Boltenkov and Güner 2020; Dalastra and Heiden 2020; Kottekkattu and Pradeep 2020; Liu et al. 2020; Naive and Sanders 2020; Nobis et al. 2020b; Wang et al. 2020; Wolski et al. 2020; Yang and Rushforth 2020). The present paper is also based on the revision of many online herbarium databases, and our main aim is the typification of five names of species described based on plant material collected by Warszewicz, which types were destroyed during World War II.

## ﻿Materials and methods

This work is based on the examination of herbarium specimens and the analysis of relevant literature (including protologues). The main source of original material of plants species collected by Warszewicz and described by Klotzsch (1851), Engler (1874), Hieronymus (1895), Schuman and Krause in Krause (1908) was herbarium of B. However, other original material of these species can be found in G, BR, K, BM, P, LD, F, NY and KRA. We reviewed plant material or used the online databases of above mentioned and some other herbaria (e.g. BGBM 2020, CRIA 2020, F 2020, MNHN 2020, NMNH 2020, NYBG 2020, S 2020, Virtual Herbaria JACQ 2020) to search for all the original material available. When there was more than one specimen or more than one gathering, we chose the most complete and best preserved one, following the description given in the protologue as well as by comparison with the photographs of the original material preserved at F as negatives. To evaluate the synonymy of typified names, we compared the plant specimens with the type specimens of the accepted species, revised the original description of these taxa and checked recent Neotropical Floras and any available taxonomic elaborations concerning particular species and genera. Names typified here are listed in alphabetic order. In some cases, we have proposed superseding some previously designated lectotypes, because they are based on photographs (Turland et al. 2018) and are therefore neotypes (Art. 9.10). The list of vouchers with specimens collected by Warszewicz, identified to the level of species or genus, and preserved in KRA, is presented in Suppl. material 1: Table S1.

Although Warszewicz’s specimens preserved in KRA have different labels in comparison with those on Macbride’s photographs, we are sure that they are duplicates of types preserved in B herbarium, because: i) some of these species and specimens still are known only from the protologue and type collection (e.g. *Esenbeckiacornuta* or *E.warscewiczii*); ii) all the specimens preserved in KRA and typified here are almost identical to those photographed by Macbride at B (they were collected in the same way and at the same phenological stage); iii) some of them have the same patterns of insect damage of leaves (e.g. *Remijiainvolucrata*). Thus, in accordance with the ICN (Turland et al. 2018) selected Warszewicz’s specimens preserved in KRA, we designate here as lectotypes.

## ﻿Results and discussion

### ﻿ Berberis warszewiczii

Taxon classificationPlantaeRanunculalesBerberidaceae

**Figure 1.**
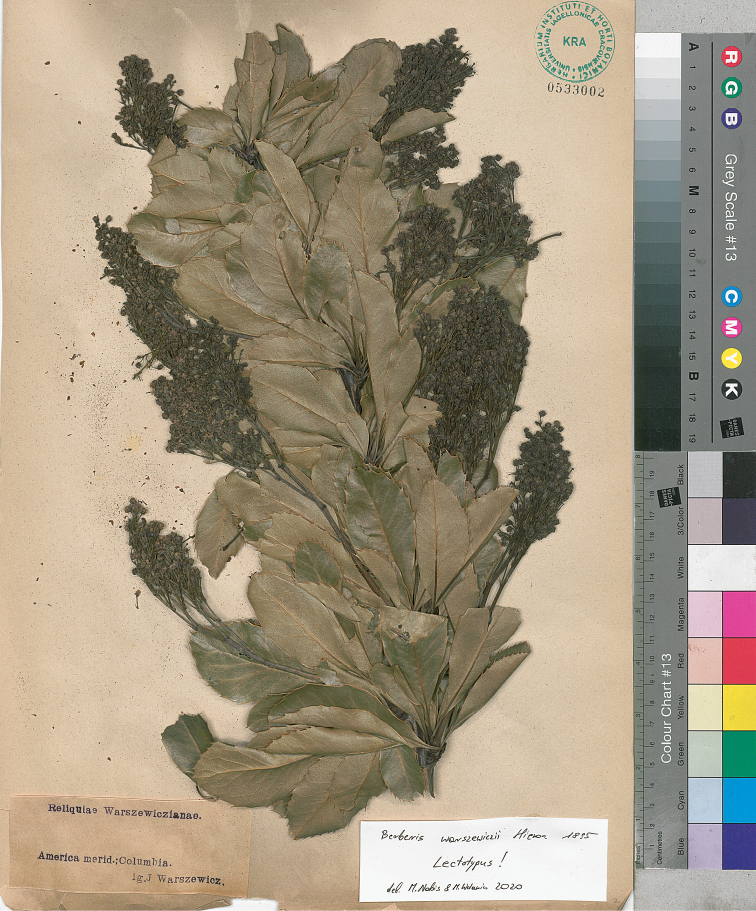
Lectotype of *Berberiswarszewiczii*.

Berberis multiflora Benth., Plantas Hartwegianas imprimis Mexicanas 124. 1843. Type Protologue: In montibus Santiago et Saraguru. Type: [EQUADOR] Mountain of Santiago & Saraguru, *Hartweg 708* (lectotype, designated here, K 407116! [Herbarium Benthamianum], isolectotypes, K 407120! [Herbarium Benthamianum], K 407118!, BM 778308!, BR 695719!, E 373177!, F 870723!, F 894660!, LD 1689638!, NY 7374!, P 752174!, P 752175!).
= Berberiswarszewiczii Hieron., Botanische Jahrbücher für Systematik, Pflanzengeschichte und Pflanzengeographie 20: beibl. 49: 13 (1895). Type Protologue: ECUADOR, prope urbeum Cuenca, *Warszewicz 4[25]*. Type: Cuenca in Equador, Süd America, *Warszewicz s.n.* (holotype, B destroyed, photograph at F! negative no. 14317, https://fm-digital-assets.fieldmuseum.org/28/661/14317.jpg; lectotype, designated here, America merid. Columbia, *J. Warszewicz s.n.* (KRA 533002!; Fig. 1), isolectotypes, KRA 533000!, KRA 533001!).

#### Remarks.

The genus *Berberis* L. (including *Mahonia* Nutt.) comprises ca. 650 species of shrubs or small trees that are not very widespread in the Northern hemisphere. In the Neotropics its distribution is limited to mountainous regions. In South America it reaches Tierra del Fuego and south-eastern Brazil. In these regions only species with simple (not compound) leaves are recorded (Ahrendt 1961; Landrum 1999; Ulloa Ulloa 2009). *Berberismultiflora* Benth. (synonyms: *B.loxensis* Benth., *B.warszewiczii* Hieron. and B.multifloravar.calvescens C.K. Schneid.; Ulloa Ulloa 1999), which is one such species, is native to Ecuador and Peru (Brako and Zarucchi 1993; Ulloa Ulloa 1999). The species is considered to be morphologically very variable. However, studying numerous type specimens (syntypes) of *B.multiflora* collected by *Hartweg 708* in Peru (one of them, mounted to the sheet with stamp Herbarium Benthamianum 1854, K 407116!, is here designated as lectotype), we noticed that they somewhat differ from these representing *B.warszewiczii* (F! negative no. 14317, https://fm-digital-assets.fieldmuseum.org/30/341/14317.jpg, KRA). The main difference between the two species concerns the leaf morphology. In the case of *B.warszewiczii*, leaf edge (excluding the base) is regularly spine-toothed while leaves of *B.multiflora* are entire, or with a few spines. However, during the revision of specimens representing *B.multiflora*, we noticed several specimens with spine-toothed leaves, what makes them more similar to *B.warszewiczii* or even to *B.pectinata*. Revision using molecular analyses is needed in this group of taxa to explain their taxonomic relationship. Bearing in mind that the type specimen of *B.warszewiczii* Hieron. at B was destroyed, we designated here the specimen collected by Warszewicz and preserved at KRA as the lectotype and the two additional (KRA 533000, KRA 533001) as the isolectotypes. The specimens preserved in KRA are duplicates of the type documented on Macbride’s photograph (F negative no. 14317, https://fm-digital-assets.fieldmuseum.org/28/661/14317.jpg), and their morphology matches with the description of the taxon.

### ﻿ Esenbeckia cornuta

Taxon classificationPlantaeDipteraStratiomyidae

Esenbeckia cornuta Engl., Flora Brasiliensis 12(2): 146 (1874). Type Protologue: Peruvia pr. Jaen. de Bracamoros, *Warscewicz*. Type: Peru, *Warszewicz s.n.* (holotype, B destroyed, photograph at F! negative no. 12512, https://fm-digital-assets.fieldmusum.org/30/341/14317.jpg; lectotype, designated by Kaastra 1982: 79, K 531234! [Herb. Benthamianum], isolectotypes, NY 51856!, KRA 533031-533048! [17 sheets]).

#### Remarks.

*Esenbeckia* Kunth is represented by ca. 40 species of shrubs or trees, distributed in America: from Mexico to north-eastern Argentina, and in the West Indies (Kaastra 1982; Wilkerson and Fairchild 1983; The Plant List 2021). One of the rarest species in this genus is *Esenbeckiacornuta*, which to date is known from the type locality in Peru, and the two sheets with specimens of that taxon preserved respectively at K (http://apps.kew.org/herbcat/getImage.do?imageBarcode=K000531234) and NY (http://sweetgum.nybg.org/science/vh/specimen-details/?irn=721187; Kaastra 1982). In the herbarium of KRA, there are, however, an additional 17 sheets with original specimens of that taxon (isolectotypes). The specimens preserved in KRA constitute together with the lectotype, one of Warszewicz’s gatherings, collected in the same manner and in the same period of time in terms of phenology, development and flowering.

### ﻿ Esenbeckia warscewiczii

Taxon classificationPlantaeDipteraStratiomyidae

**Figure 2.**
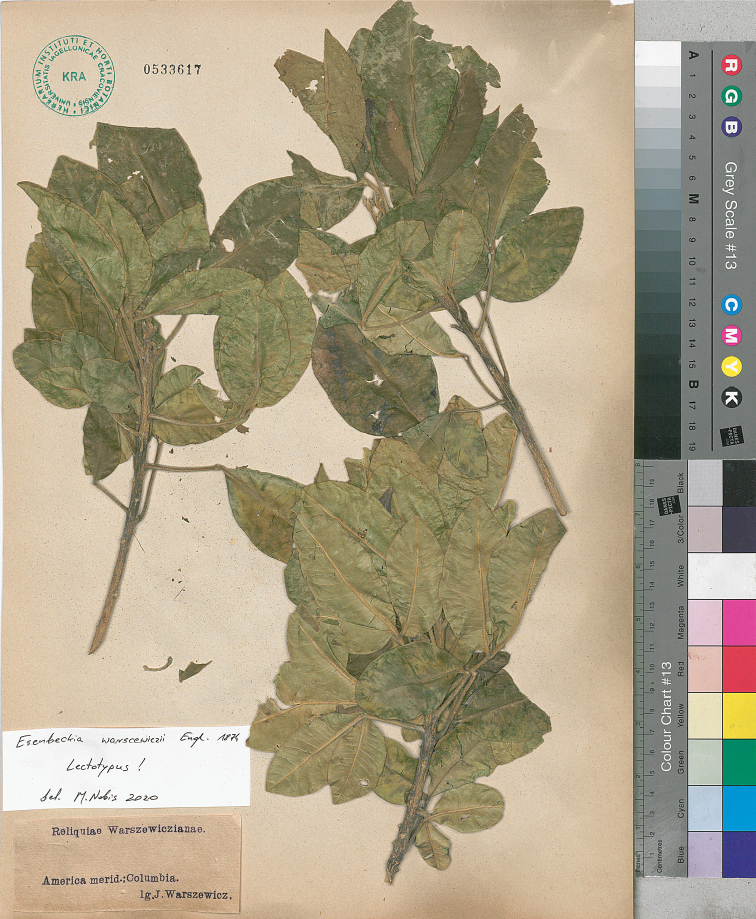
Lectotype of *Esenbeckiawarscewiczii*.

Esenbeckia warscewiczii Engl., Flora Brasiliensis 12(2): 148 (1874). Type Protologue: [Peru] Peruvia boreali in viculo Sonda, *Warscewicz*. Type: Peru, *Warszewicz s.n.* (holotype, B destroyed, photograph at F! negative no. 12519, https://fm-digital-assets.fieldmuseum.org/28/668/12519.jpg; lectotype, designated here, America merid. Columbia, *J. Warszewicz s.n.* (KRA 533617!; Fig. 2).

#### Remarks.

Although the specimens of *Esenbeckiawarscewiczii* preserved in KRA have no flowers and fruits, they completely match the description of the taxon (Engler 1874; Kaastra 1982). We have no doubt that the specimens in KRA and type documented by Macbride (photograph at F!) constitute one gathering.

### ﻿ Proclesia veraguensis

Taxon classificationPlantaeEricalesEricaceae

Cavendishia bracteata (Ruiz & Pav. ex J. St.-Hil.) Hoerold, Botanische Jahrbücher für Systematik, Pflanzengeschichte und Pflanzengeographie 42(4): 280 (1909).
≡ Thibaudiabracteata Ruiz & Pav. ex J. St.-Hil., Exposition des Familles Naturelles 1(2): 363. 1805. Type Protologue: Peru. Huanuco: “In Peruviae Andium montibus altis frigidis, inter saxa, et argillosis locis and Pillao tractus,” 1778–1788, *Ruiz & Pavón s.n.* (lectotype, designated by Luteyn 1983:137, MA-15/54, photo NY neg. 9256).
= Proclesiaveraguensis Klotzsch, Linnaea 24: 35 (1851). Type Protologue: In Costa Rica, *de Warszewicz s.n.* Type: Costa Rica et Veragua, *Warszewicz s.n.* (holotype, B destroyed; neotype, designated by Luteyn (1983: 140), photograph at F! negative no. 4682, http://ww2. bgbm.org/herbarium/images/FieldMuseum/4682.jpg).
≡ Cavendishiaveraguensis (Klotzsch) Hemsl., Biologia Centrali-Americana, Botany 2(10): 273 (1881)
≡ Chupalonveraguense (Klotzsch) Kuntze, Revisio Generum Plantarum 2: 383 (1891).

#### Remarks.

The genus *Cavendishia* comprises more than 100 species of neotropical shrubs, having bright, snowy flowers, usually enlarged floral bracts, and alternately unequal filaments and anthers. The genus occurs mostly in the mountains of the north-western part of South America, especially in Colombia. Most species representing the genus *Cavendishia* are narrow endemics, and only a few of them are widespread (Luteyn 1983). One of the widespread species is *C.bracteata*, which appears to be a morphologically extremely variable taxon. Luteyn (1983) listed a number of species which are suspected to be conspecific with *C.bracteata*. One of them is *Proclesiaveraguensis* described by Klotzsch (1851). Because Klotzsch did not mention either the collection number or the place of preservation of the holotype, and the possibility that the original material of that taxon was destroyed during WWII, Luteyn (1983: 140) designated a photograph of original specimens (in ACS neg. 13 and F neg. 4682) as a lectotype of the taxon. However, bearing in mind, that according to Art. 9.10 of the ICN (Turland et al. 2018) such photographs cannot be chosen as lectotypes thus it is here corrected to a neotype of *Proclesiaveraguensis*.

In the KRA herbarium, there are three sheets with specimens of *Cavendishia* collected by Warszewicz (America merid. Columbia, *J. Warszewicz s.n.*, KRA 533506, KRA 533507, KRA 533508), however, identifying whether they are conspecific with *Proclesiaveraguensis* or not, requires further studies.

### ﻿ Psammisia ramiflora

Taxon classificationPlantaeEricalesEricaceae

Psammisia ramiflora Klotzsch, Linnaea 24: 44 (1851). Type Protologue: In locis alpestribus Veraguae Americae centralis, *de Warszewicz*. Type: [label 1] Costa Rica et Veragua Chiriqui, [label 2] Zentral America, de Warszewicz s.n. (holotype, B destroyed, photograph at F! negative no. 4698, https://fm-digital-assets.fieldmuseum.org/26/134/4698.jpg; lectotype, labeled by Leutyn in 2006 and designated here, Costa Rica and Veragua, *Warszewicz s.n.* G 352123!).

#### Remarks.

*Psammisia* is a Neotropic genus of terrestrial or epiphytic shrubs from the Ericaceae family, consisting of ca. 50 species. Its distribution ranges from Costa Rica southward into Bolivia and eastward to French Guiana and Trinidad (Wilbur and Luteyn 1978; The Plant List 2021). Within the genus, one of the most variable morphologically is *Psammisiaramiflora*, known only from Costa Rica and Panama. The taxon was described by Klotzsch (1851) based on Warszewicz’s gathering, destroyed during the war. During our studies, we found only one specimen collected by Warszewicz and determined by Klotzsch as *P.ramiflora*. The specimen is preserved in G herbarium. However, its label slightly differs from both, specified in the protologue and on the type specimen photographed by Macbride (F negative no. 4698). Nevertheless, it constitutes a duplicate of the holotype. In 2006, Luteyn labelled this specimen as the lectotype of the species; however, he did not designate it in publication. Thus, we here propose to designate the specimen preserved in G (https://www.ville-ge.ch/musinfo/bd/cjb/chg/adetail.php?id=238797&base=img&lang=en) as the lectotype of *P.ramiflora*.

### ﻿ Remijia involucrata

Taxon classificationPlantaeGentianalesRubiaceae

**Figure 3.**
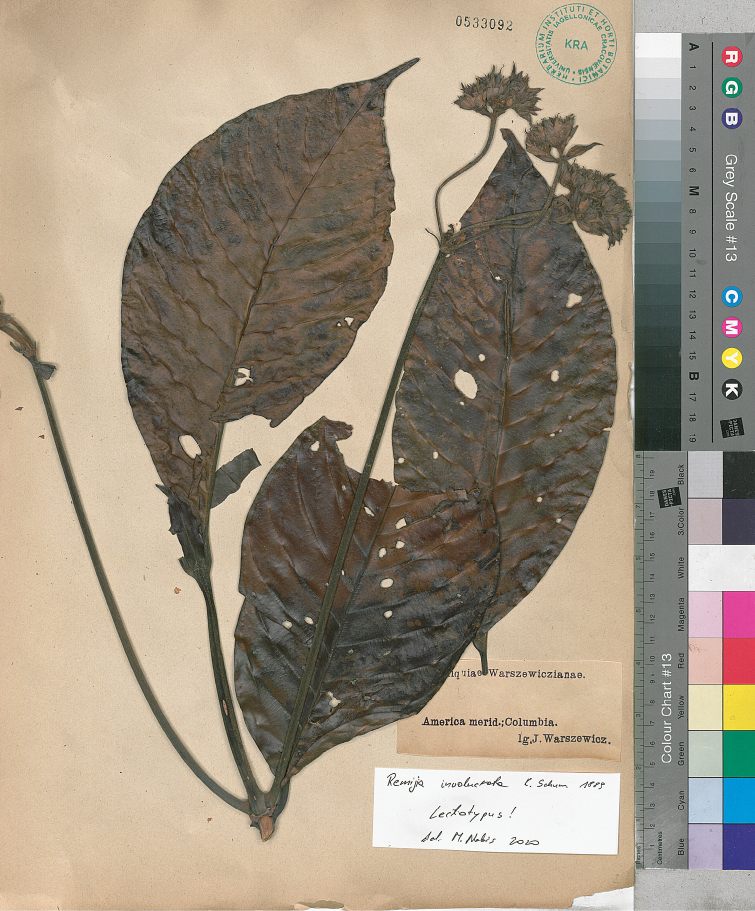
Lectotype of *Remijiainvolucrata*.

Ciliosemina purdieana (Wedd.) A. Antonelli, Taxon 54(1): 26 (2005).
≡ Remijiapurdieana Wedd., Ann. Sci. Nat. 11: 272. 1849. Type Protologue: Colombia, Antioquia, Cauvas, s.d., *Purdie s.n.* (holotype, P 1900374!).
= Remijiainvolucrata K. Schum., Flora Brasiliensis 6(6): 150 (1889). Type Protologue: [New Grenada] in ditione Novo-Granatensis, *Warszewicz s.n.* Type: Neu-Granada: West Cordillere, 4–6000’, *v. Warszewicz s.n.* (holotype, B destroyed, photograph at F! negative no. 165, https://fm-digital-assets.fieldmuseum.org/21/591/165.jpg; lectotype, designated here, America merid. Columbia, *J. Warszewicz s.n.* (KRA 533092!; Fig. 3), isolectotypes KRA 533080-533091! [12 sheets]).

#### Remarks.

Following Andersson and Antonelli (2005)*Cilioseminapurdieana* (syn. *Remijiapurdieana*) is a narrow endemic, occurring in the lower section of the Magdalena River Valley in the Colombian departments of Antioquia, Bolívar, and Santander. To the synonyms of this taxon belong also *Remijiainvolucrata*, determined by J. Klotzsch (name on herbarium sheet in B, F! negative no. 165) and described over 30 years later by Schumann (1889). Because the holotype of *R.involucrata* was destroyed, we designated here as the lectotype the specimen collected by Warszewicz and preserved in KRA (Fig. 3). The remaining 12 specimens of the taxon in KRA are isolectotypes.

### ﻿ Rondeletia orthoneura

Taxon classificationPlantaeBeryciformesRondeletiidae

**Figure 4.**
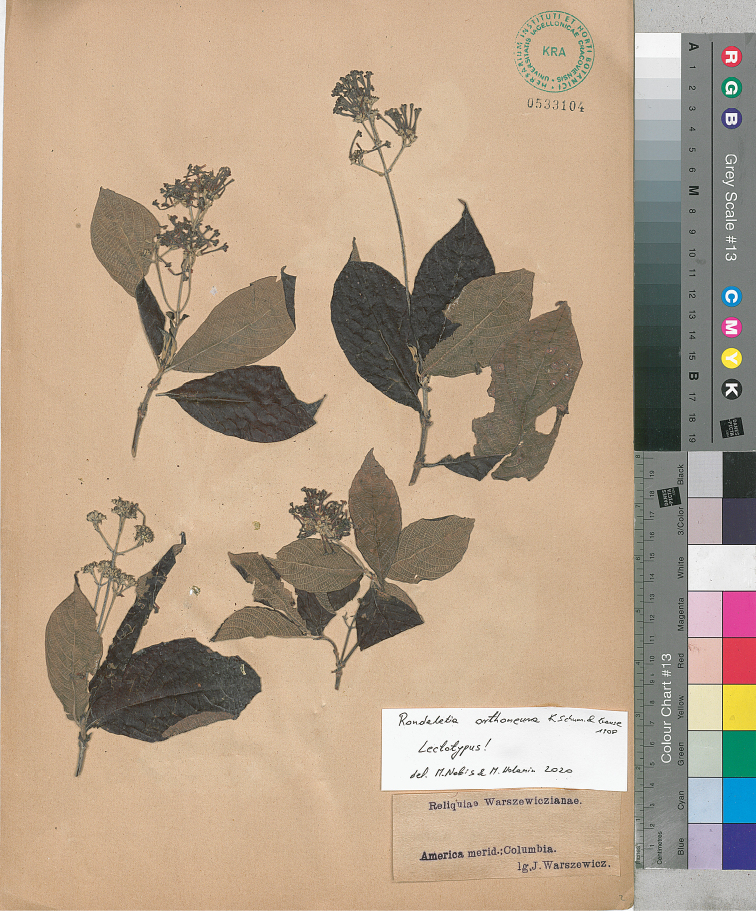
Lectotype of *Rondeletiaorthoneura*.

Arachnothryx reflexa (Benth.) Planch., Flore des Serres et des Jardins de l’Europe 5: 442. 1849.
≡ Rondeletiareflexa Benth., Plantas Hartwegianas imprimis Mexicanas 192. 1839. Type: Prope pagum Villeta, prov. Bogota, *Hartweg 1052* (lectotype, designated here, K 174038! [herbarium Benthamianum], isolectotypes, K 174517!, BR 5267125!, BR 5267095!, F 766934!, F 871798!, P 3906070!, P 3906069!, LD 1211768!, B destroyed – photograph at F! negative no. 89, http://ww2.bgbm.org/herbarium/images/FieldMuseum/87.jpg, negative no. 25758, https://fm-digital-assets.fieldmuseum.org/1542/161/25758.jpg).
= Rondeletiaorthoneura K. Schum. & Krause in Krause, Botanische Jahrbücher für Systematik, Pflanzengeschichte und Pflanzengeographie 40: 314–315. 1908. Type Protologue: In Columbia: sine loco, *v. Warszewicz 622*. Type: *Warszewicz s.n.* (holotype, B destroyed; photograph at F! negative no. 89, http://ww2.bgbm.org/herbarium/images/FieldMuseum/89.jpg; lectotype, designated here, America merid. Columbia, *J. Warszewicz s.n.* (KRA 533104!; Fig. 4), isolectotype, KRA 533105!).

#### Remarks.

Because the specimens representing *R.orthoneura*, collected by Warszewicz and preserved at B, were destroyed during WWII, we designated the specimen of *R.orthoneura* (in KRA), as the lectotype for the taxon. According to Lorence (2012) and Borhidi (2017), *Rondeletiaorthoneura* is conspecific with *Rondeletiainconstans* Standl., Publications of the Field Museum of Natural History, Botanical Series 7: 31 (1930). Type: Colombia, Quetame, Pipiral, eastern Andes de Bogotá, alt. 1000–1600 m., July 1897, *F.C. Lehmann 8751* (holotype F!) = Arachnothryxreflexavar.inconstans (Standl.) Steyerm. Among numerous type specimens (syntypes) of *R.reflexa* collected by *Hartweg 1052* in Bogota, the one, mounted to the sheet with stamp Herbarium Benthamianum 1854 (K 174038!), is designated here as the lectotype.

#### Notes.

It is worth noting, that in the KRA herbarium there are several sheets with specimens (duplicates of specimens destroyed in B) named by J. Klotzsch but never described (nomina nuda). These include *Rondeletiaferruginea* Klotzsch (https://fm-digital-assets.fieldmuseum.org/21/689/26.jpg; KRA 533668!), *Ladenbergiawarscewicziana* Klotzsch (https://fm-digital-assets.fieldmuseum.org/21/557/133.jpg; KRA 533185–533197! [13 sheets]), and *Rustiawarscewicziana* Klotzsch (https://fm-digital-assets.fieldmuseum.org/21/532/11.jpg, KRA 533663–533667! [5 sheets]). The latter taxon was identified, based on Macbride photographs, as *Rustiathibaudioides* (H. Karst.) Delprete by Delprete (1999). The other taxa require further studies.

## Supplementary Material

XML Treatment for Berberis warszewiczii

XML Treatment for Esenbeckia cornuta

XML Treatment for Esenbeckia warscewiczii

XML Treatment for Proclesia veraguensis

XML Treatment for Psammisia ramiflora

XML Treatment for Remijia involucrata

XML Treatment for Rondeletia orthoneura

## Supplementary materials

Supplementary material 1 Table S1 Data type list of herbarium vouchers Explanation note The list of vouchers with specimens collected by Warszewicz preserved in KRA. https://binary.pensoft.net/file/657033This dataset is made available under the Open Database License (http://opendatacommons.org/licenses/odbl/1.0/). The Open Database License (ODbL) is a license agreement intended to allow users to freely share, modify, and use this Dataset while maintaining this same freedom for others, provided that the original source and author(s) are credited.Marcin Nobis, Ewelina Klichowska, Mateusz Wolanin, Agnieszka Nobis, Arkadiusz Nowak

## References

[B1] Ahrendt LWA (1961) *Berberis* and *Mahonia*: A taxonomic revision. Botanical Journal of the Linnean Society 57(369): 1–410. 10.1111/j.1095-8339.1961.tb00889.x

[B2] Alves-Araújo A dos Santos Moraes Q Nichio-Amaral R Miranda VS (2020) Typifications in neotropical Sapotaceae. PhytoKeys 170: 45–69. 10.3897/phytokeys.170.54718 33442323 PMC7769886

[B3] Andersson L Antonelli A (2005) Phylogeny of the tribe Cinchoneae (Rubiaceae), its position in Cinchonoideae, and description of a new genus, *Ciliosemina*. Taxon 54(1): 17–28. 10.2307/25065299

[B4] Bebber DP Carine MA Wood JRI Wortley AH Harris DJ Prance GT Davidse G Paige J Pennington TD Robson NKB Scotland RW (2010) Herbaria are a major frontier for species discovery. Proceedings of the National Academy of Sciences of the United States of America 107(51): 22169–22171. 10.1073/pnas.1011841108 21135225 PMC3009773

[B5] BGBM (2020) Herbarium Berolinense Virtual. http://ww2.bgbm.org/herbarium/ [accessed 22 October 2020]

[B6] Boltenkov EV Güner A (2020) Typification of some *Oncocyclus* (Iris, Iridaceae) names related to the Turkish flora. Phytotaxa 468(1): 045–061. 10.11646/phytotaxa.468.1.2

[B7] Borhidi AL (2017) Revisión crítica del género Arachnothryx Planch. Acta Botanica Hungarica 59(3–4): 287–318. 10.1556/034.59.2017.3-4.2

[B8] Borsch T Stevens AD Häffner E Güntsch A Berendsohn WG Appelhans M Barilaro C Beszteri B Blattner F Bossdorf O Dalitz H Dressler S Duque-Thüs R Esser H-J Franzke A Goetze D Grein M Grünert U Hellwig F Hentschel J Hörandl E Janßen T Jürgens N Kadereit G Karisch T Koch M Müller F Müller J Ober D Porembski S Poschlod P Printzen C Röser M Sack P Schlüter P Schmidt M Schnittler M Scholler M Schultz M Seeber E Simmel J Stiller M Thiv M Thüs H Tkach N Triebel D Warnke U Weibulat T Wesche K Yurkov A Zizka G (2020) A complete digitization of German herbaria is possible, sensible and should be started now. Research Ideas and Outcomes 6: e50675. 10.3897/rio.6.e50675

[B9] Brako L Zarucchi JL (1993) Catalogue of the flowering plants and gymnosperms of Peru: Catálogo de las angiospermas y gimnospermas del Perú. Monographs in systematic botany from the Missouri Botanical Garden 45: 1–1286.

[B10] CRIA (2020) (Centro de Referência e Informação Ambiental). Specieslink-simple search. http://www.splink.org.br/index [accessed 28 August 2020]

[B11] Dalastra CH Heiden G (2020) Typifications of five names in *Agarista* (Ericaceae, Vaccinioideae, Lyonieae). Embrapa Clima Temperado-Artigo em periódico indexado (ALICE). Phytotaxa 474(2): 179–184. 10.11646/phytotaxa.474.2.8

[B12] Delprete PG (1999) Rondeletieae (Rubiaceae): Part I (*Rustia*, *Tresanthera*, *Condaminea*, *Picardaea*, *Pogonopus*, *Chimarrhis*, *Dioicodendron*, *Molopanthera*, *Dolichodelphys*, and *Parachimarrhis*). Flora Neotropica, 225 pp.

[B13] Engler A (1874) Rutaceae. In: Martius CFP Eichler AG (Eds) Flora brasiliensis, vol.12, pt.2. Frid. Fleischer, Lepzig, Munich, 75–196.

[B14] FMBC (2020) Field Museum Botanical Collections. https://collections-botany.fieldmuseum.org/list [accessed 12 October 2020]

[B15] Funk V (2003) The importance of herbaria. Plant Science Bulletin 49: 94–95.

[B16] Harris KM Marsico TD (2017) Digitizing specimens in a small herbarium: A viable workflow for collections working with limited resources. Applications in Plant Sciences 5(4): e1600125. 10.3732/apps.1600125 PMC5400430 28439474

[B17] HC (S) (2020) Herbarium Catalogue (S). http://herbarium.nrm.se/search/specimens/ [accessed 15 October 2020]

[B18] Henning T Plitzner P Güntsch A Berendsohn W Müller A Kilian N (2018) Building compatible and dynamic character matrices – Current and future use of specimenbased character data. Botany Letters 165(3–4): 352–360. 10.1080/23818107.2018.1452791

[B19] Hiepko P (1987) The collections of the Botanical Museum Berlin-Dahlem (B) and their history. Englera 7: 219–252.

[B20] Hieronymus G (1895) PlantaeLehmannianae in Guatemala, Costarica, Columbia et Ecuador collectae, additis quibusdam ad aliis collectoribus ex iisdem regionibus necnone Venezuela et Peruvia allatis, quas determinavit et descripsit adjuvantibus aliis auctoribus. Botanische Jahrbücher für Systematik, Pflanzengeschichte und Pflanzengeographie 20(49): 1–72.

[B21] IPNI (2021) International Plant Names Index. https://www.ipni.org/ [accessed 12 January 2021]

[B22] Jungfer K-H (2017) On Warszewicz’s trail: The identity of *Hylamolitor* O. Schmidt. Salamandra (Frankfurt) 53(1): 18–24.

[B23] Kaastra RC (1982) A monograph of the Pilocarpinae (Rutaceae). Flora Neotropica 33: 1–197.

[B24] Klotzsch JF (1851) Studien über die natürliche Klasse Bicornes Linné. Linnaea 24: 1–88.

[B25] Köhler P (2014) The life of Józef Warszewicz (1812–1866): The Kraków period. Acta Baltica Historiae et Philosophiae Scientiarum 2(1): 18–36. 10.11590/abhps.2014.1.02

[B26] Kottekkattu T Pradeep AK (2020) Lectotypification and a new record of the genus *Eragrostiella* (Tripogoninae, Cynodonteae, Chloridoideae, Poaceae) from India. Phytotaxa 464(1): 109–115. 10.11646/phytotaxa.464.1.10

[B27] Krause K (1908) Rubiaceae andinae. Botanische Jahrbücher für Systematik, Pflanzengeschichte und Pflanzengeographie 40: 312–351.

[B28] Landrum L (1999) Revision of *Berberis* (Berberidaceae) in Chile and adjacent southern Argentina. Annals of the Missouri Botanical Garden 86(4): 793–834. 10.2307/2666170

[B29] Lang PLM Willems FM Scheepens JF Burbano HA Bossdorf O (2019) Using herbaria to study global environmental change. The New Phytologist 221(1): 110–122. 10.1111/nph.15401 30160314 PMC6585664

[B30] Liu BB Liu GN Hong DY Wen J (2020) Typification of 23 names in *Eriobotrya* (Maleae, Rosaceae). PhytoKeys 139: 99–118. 10.3897/phytokeys.139.47967 32089637 PMC7012954

[B31] Lorence DH (2012) *Arachnothryx Planch*. In: Davidse G, Sousa MS, Knapp SY, Chiang F (Eds) Flora Mesoamericana 4(2): 16–37.

[B32] Luteyn JL (1983) Ericaceae: Part I. *Cavendishia*. Flora Neotropica 35: 1–289.

[B33] Meineke E Davis C Davies J (2018) The unrealised potential of herbaria for global change biology. Ecological Monographs 88(4): 505–525. 10.1002/ecm.1307

[B34] MNHN (2020) Muséum National d’Histoire Naturelle. https://science.mnhn.fr/all/search/form [accessed 18 October 2020]

[B35] Naive MAK Sanders T (2020) Typification of two species names in *Dendrochilum* (Orchidaceae, subgenus Platyclinis). Phytotaxa 470(4): 298–299. 10.11646/phytotaxa.470.4.4

[B36] Nascimento Jr JE Bittrich V Amaral MCE (2019) Typification of names of Clusiaceae based on material collected by August Weberbauer in Peru. Willdenowia 49(2): 193–196. 10.3372/wi.49.49208

[B37] NMNH (2020) Smithsonian National Museum of Natural History online database. https://collections.nmnh.si.edu/search/botany/ [accessed 25 August 2020]

[B38] Nobis M Głuszak T Zemanek A Zemanek B (2020a) Lectotypication of *Warszewicziapulcherrima* (Rubiaceae) with note on Józef Warszewicz’s plant collection preserved at KRA herbarium from his trip to Central and South America. Phytotaxa 442(1): 47–51. 10.11646/phytotaxa.442.1.9

[B39] Nobis M Gudkova PD Nowak A Sawicki J Nobis A (2020b) A synopsis of the genus *Stipa* (Poaceae) in Middle Asia, including a key to species identification, an annotated checklist, and phytogeographic analyses. Annals of the Missouri Botanical Garden 105(1): 1–63. 10.3417/2019378

[B40] NYBG (2020) C. V. Starr Virtual Herbarium. http://sweetgum.nybg.org/science/vh/ [accessed 30 October 2020]

[B41] Sauter CO (2010) Josef Ritter Von Rawicz Warscewicz (1812–1866). Biocenosis 23: 56–61.

[B42] Savage JM (1970) On the trail of the golden frog: with Warscewicz and Gabb in Central America. Proceedings of the California Academy of Sciences, Fourth Series 38: 273–288.

[B43] Schumann C (1889) Rubiaceae. Tribus Naucleeae, Henriquezieae, Cinchoneae, Rondeletieae, Condamineeae, Hedyotideae, Mussaendeae, Catesbaeeae, Hamelieae, Gardenieae. In: Martius CFP de, Eichler AG, Urban I (Eds) Flora Brasiliensis. Enumeratio plantarum in Brasilia hactenus detectarum quas suis aliorumque botanicorum studiis descriptas et methodo naturali digestas partim icone illustratas, 6(6), Frid. Fleischer, Lipsiae [Leipzig], 125–442; 94–151.

[B44] Staples G Prado J (2018) Clarification is needed in the Code for the nomenclatural status of type specimen photographs. Taxon 67(5): 833–835. 10.12705/675.2

[B45] Sukhorukov AP Liu PL Kushunina M (2019) Taxonomic revision of Chenopodiaceae in Himalaya and Tibet. PhytoKeys 116: 1–141. 10.3897/phytokeys.116.27301 PMC6367307 30740023

[B46] The Plant List (2021) Version 1.1. 2013. http://www.theplantlist.org/ [accessed 16 January 2021]

[B47] Thiers B (2021 [continuously updated]) Index Herbariorum. A global directory of public herbaria and associated staff. New York Botanical Garden’s Virtual Herbarium. http://sweetgum.nybg.org/science/ih [accessed 10 January 2021]

[B48] Turland NJ Wiersema JH Barrie FR Greuter W Hawksworth DL Herendeen PS Knapp S Kusber W-H Li D-Z Marhold K May TW McNeill J Monro AM Prado J Price MJ Smith GF (2018) International Code of Nomenclature for algae, fungi, and plants (Shenzhen Code) adopted by the Nineteenth International Botanical Congress Shenzhen, China, July 2017. Regnum Vegetabile 159. Koeltz Botanical Books, Glashütten. 10.12705/Code.2018

[B49] Ulloa Ulloa C (1999) Berberidaceae. In: Jørgensen PM León-Yánez S (Eds) Catalogue of Vascular plants of Ecuador.Monographs in Systematic Botany from the Missouri Botanical Garden 75: 319–320.

[B50] Ulloa Ulloa C (2009) Neotropical Berberidaceae. In: Milliken W, Klitgård B, Baracat A (2009 onwards), Neotropikey – Interactive key and information resources for flowering plants of the Neotropics. http://www.kew.org/science/tropamerica/neotropikey/families/Berberidaceae.htm

[B51] Virtual Herbaria JACQ (2020) Virtual Herbaria JACQ. https://herbarium.univie.ac.at/database/search.php [accessed 28 October 2020]

[B52] Wang W Su Z Ma Z (2020) Lectotypification of five names in the genus *Stellaria* (Caryophyllaceae) in China. PhytoKeys 170: 71–81. 10.3897/phytokeys.170.59527 33442324 PMC7769891

[B53] Wilbur RL Luteyn JL (1978) Flora of Panama. Part VIII. Family 149. Ericaceae. Annals of the Missouri Botanical Garden 65(1): 27–143. 10.2307/2395354

[B54] Wilkerson RC Fairchild GB (1983) A review of the South American species of EsenbeckiasubgenusEsenbeckia (Diptera: Tabanidae). Journal of Natural History 17(4): 519–567. 10.1080/00222938300770451

[B55] Wolski GJ Faltyn-Parzymska A Proćków J (2020) Lectotypification of the name *Stereodonnemoralis* Mitt. (Plagiotheciaceae), a basionym of *Plagiotheciumnemorale* (Mitt.) A. Jaeger. PhytoKeys 155: 141–153. 10.3897/phytokeys.155.51469 32863725 PMC7428462

[B56] Yang Y Rushforth K (2020) Lectotypification of *Abiesfanjingshanensis* (Pinaceae). PhytoKeys 152: 105–110. 10.3897/phytokeys.152.51494 32733135 PMC7360631

[B57] Zemanek A (2013) Łowca roślin Józef Warszewicz (1812–1866) – w 200-lecie urodzin. The Alma Mater 2012/2013(152–153): 43–46. [Plant hunter Józef Warszewicz (1812–1866) – on the 200^th^ anniversary of his birth]

[B58] Zemanek A Zemanek B Głuszak T Nobis M (2021) Józef Warszewicz (1812–1866) and *Warszewicziacoccinea* – a popular ornamental plant from the American tropics. Studia Historiae Scientiarum 20: 601–625. 10.4467/2543702XSHS.21.017.14048

